# Patterns and determinants of elephant attacks on humans in Nepal

**DOI:** 10.1002/ece3.7796

**Published:** 2021-08-01

**Authors:** Ashok Kumar Ram, Samrat Mondol, Naresh Subedi, Babu Ram Lamichhane, Hem Sagar Baral, Laxminarayanan Natarajan, Rajan Amin, Bivash Pandav

**Affiliations:** ^1^ Wildlife Institute of India (WII) Dehradun India; ^2^ Department of National Parks and Wildlife Conservation (DNPWC) Kathmandu Nepal; ^3^ National Trust for Nature Conservation (NTNC) Lalitpur Nepal; ^4^ Zoological Society of London (ZSL) ‐ Nepal Kathmandu Nepal; ^5^ Zoological Society of London (ZSL) London UK

**Keywords:** Asian elephant, attacks on humans, spatiotemporal pattern, Terai–Chure region

## Abstract

Attacks on humans by Asian elephant (*Elephas maximus*) is an extreme form of human–elephant conflict. It is a serious issue in southern lowland Nepal where elephant‐related human fatalities are higher than other wildlife. Detailed understanding of elephant attacks on humans in Nepal is still lacking, hindering to devising appropriate strategies for human–elephant conflict mitigation. This study documented spatiotemporal pattern of elephant attacks on humans, factors associated with the attacks, and human/elephant behavior contributing to deaths of victims when attacked. We compiled all the documented incidences of elephant attacks on humans in Nepal for last 20 years across Terai and Chure region of Nepal. We also visited and interviewed 412 victim families (274 fatalities and 138 injuries) on elephant attacks. Majority of the victims were males (87.86%) and had low level of education. One fourth of the elephant attacks occurred while chasing the elephants. Solitary bulls or group of subadult males were involved in most of the attack. We found higher number of attacks outside the protected area. People who were drunk and chasing elephants using firecrackers were more vulnerable to the fatalities. In contrast, chasing elephants using fire was negatively associated with the fatalities. Elephant attacks were concentrated in proximity of forests primarily affecting the socioeconomically marginalized communities. Integrated settlement, safe housing for marginalized community, and community grain house in the settlement should be promoted to reduce the confrontation between elephants and humans in entire landscape for their long‐term survival.

## Graphical abstract

We compiled all the documented incidences of elephant attacks on humans in Nepal for last 20 years across elephant range in Nepal. The study documented spatiotemporal pattern of elephant attacks on humans, factors associated with the attacks, and human–elephant behavior contributing to deaths of victims when attacked. We documented that elephant attacks were concentrated in proximity of forests primarily affecting the socioeconomically marginalized communities.

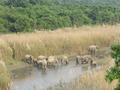

## 1 INTRODUCTION

**FIGURE 1:**
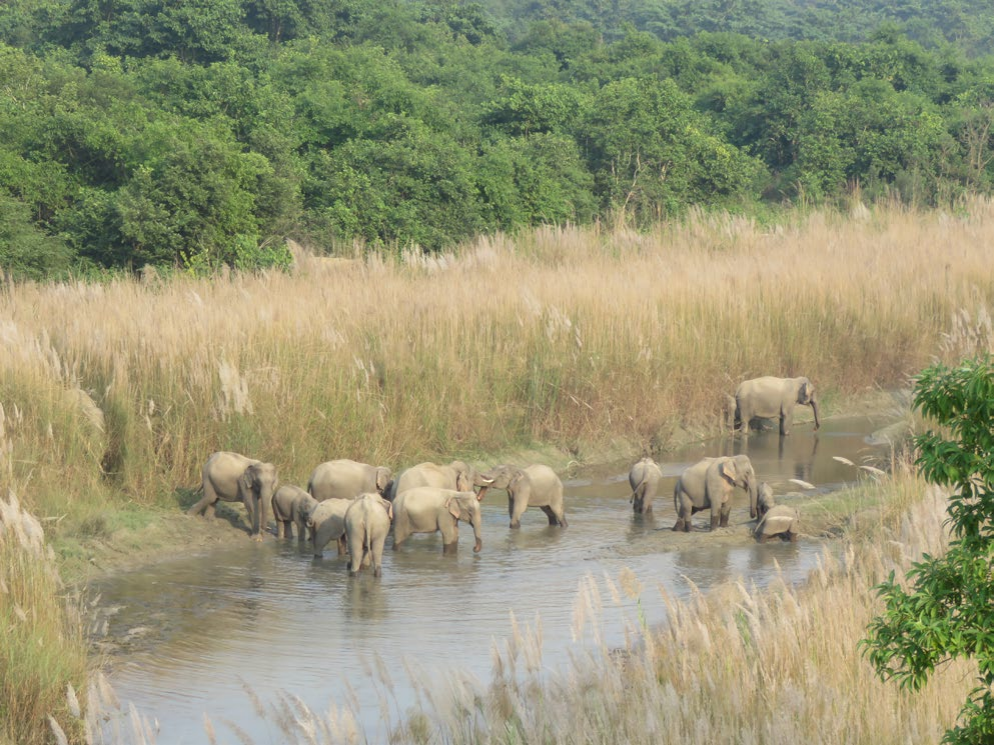
Elephants in Bardiya National Park (Photo credit: Naresh Subedi)

Asian elephant (*Elephas maximus*, referred to as “elephant” hereafter; Figure 1) is a globally endangered megaherbivore (Williams et al., 2020). It is an umbrella species in tropical and subtropical forests of Asia and has a strong cultural role in various Asian societies (Jadhav & Barua, 2012; Menon et al., 1996; Sukumar, 2003; Vasudev et al., 2020). Once widely distributed in Asia, elephants are now confined to ca. 5% of their historical range in highly fragmented landscapes (Sukumar, 2006). In addition, the rapid development of linear infrastructures including railways, highways, electric transmission lines and irrigation canals cause further obstruction to elephant movement. Elephants require large areas for their survival with long‐distance seasonal movements (Goswami, 2017; Leimgruber et al., 2003). However, increasing habitat fragmentation brings them in frequent confrontation with humans. As a result, human–elephant conflict (HEC) is escalating and has become a prominent cause of elephant population decline (Sukumar, 2006). Attack on humans by elephants is the extreme form of HEC. Other effects upon local people from HEC include loss of crops, damage to property, and safety threats (Dickman, 2010; Gross et al., 2021); and a large number of elephants are also killed in retaliation.

Nepal is a typical example of an elephant range country with a small but growing population of >200 elephants in highly fragmented landscape (Ram & Acharya, 2020). Increasing encroachment and forest conversion in the southern lowlands of the Terai and Chure (Himalayan foothills) region have destroyed the traditional migratory routes of the elephants (Ram, 2014). Whereas a few solitary bulls living in protected areas are habituated to visiting agricultural areas for a higher quality diet causing a huge amount of fiscal losses (Koirala et al., 2016). Elephants cause the highest number of human deaths among the wildlife species in Nepal. Due to this, HEC is a serious issue throughout the lowland Nepal (Acharya et al., 2016).

Few studies on human–elephant conflict have been carried out in Nepal primarily focusing on crop and property damage (Graham et al., 2016; Gross et al., 2021; Neupane et al., 2013; Pant et al., 2016). However, detailed studies of elephant attacks on humans are still lacking. This study attempts to document spatiotemporal pattern of elephant attacks on humans in Nepal, characteristics of the victims, and attacking elephants, determine factors associated with the attacks, and identify human and elephant behavior contributing to deaths of victims when attacked. We tested hypothesis: (a) Human activities are responsible for elephant attacks on humans; (b) encounters leading to attacks by elephants are higher in the proximity to forests, (c) elephant attacks were higher inside protected areas, and (d) solitary bull elephants are responsible for attacks on humans. The study results have long‐term implications for the conservation and management of elephants in the human‐dominated landscape of Nepal and beyond.

## 2 MATERIAL AND METHODS

### 2.1 Study area

**FIGURE 2:**
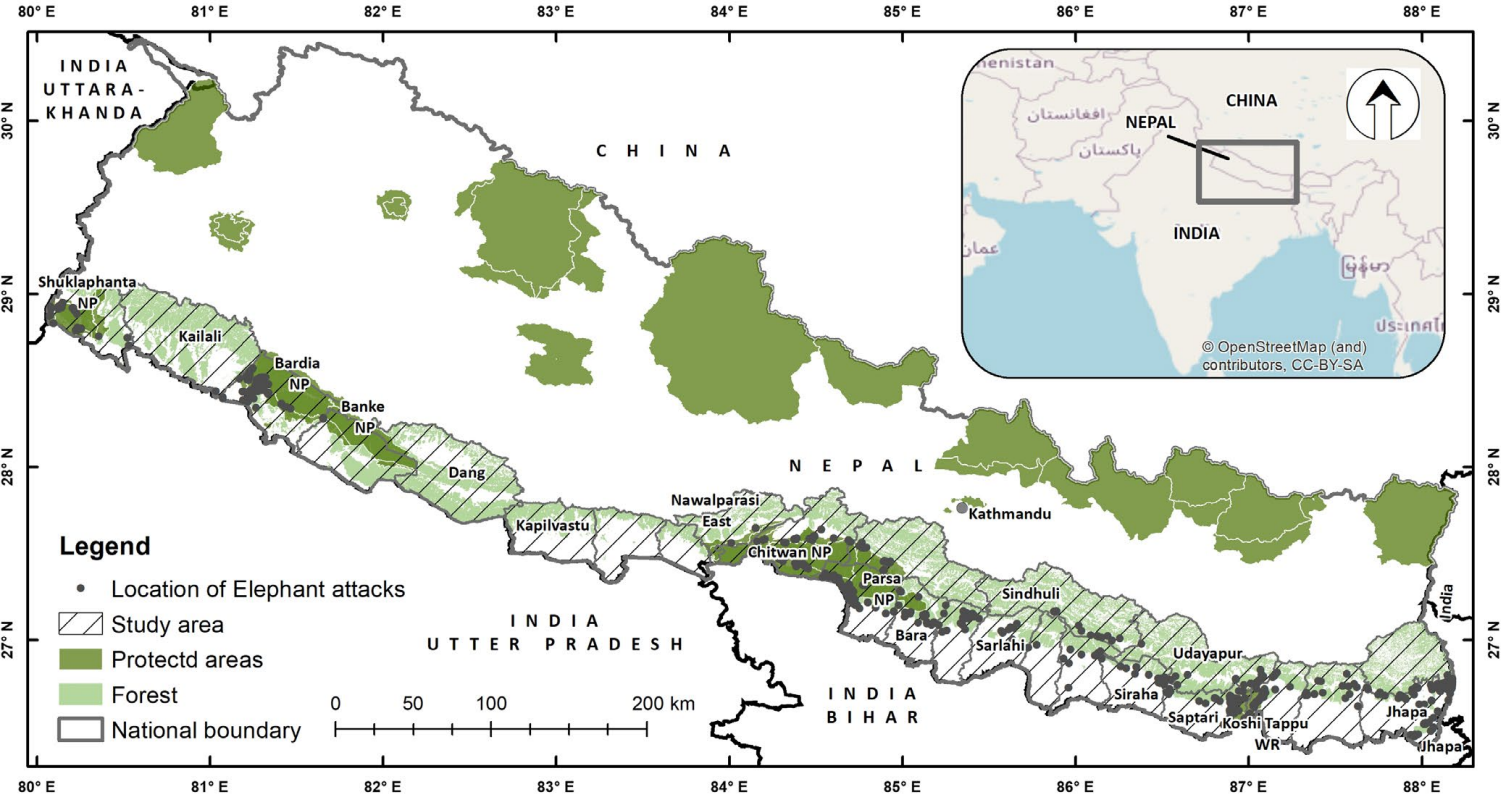
Study area location, forest cover, protected areas, and locations of elephant attacks on humans in Nepal

The study was conducted across the Terai and Chure region of Nepal covering ca. 46,000 km^2^ of elephant range in 24 districts (Figure 2). The Terai and Chure region is densely populated with 391.5 persons/km^2^ (CBS, 2012). About 51% of total population of Nepal reside in the region with agriculture and livestock husbandry as the primary occupation. About 42% of the study area is forested providing habitats and migration corridors for the elephants (DFRS, 2015). Major cities, industrial areas, and highways fragment the forested areas. The forests in the region were intact till 1950s but afterward it is under continuous human pressure from expansion of agriculture, settlements, and built‐up areas.

The study area comprises various habitats including highly productive alluvial floodplain grasslands, riverine forests, and climax sal (*Shorea robusta*) forest supporting many rare and globally threatened species including tiger (*Panthera tigris*), dhole (*Cuon alpinus*), and greater one‐horned rhinoceros (*Rhinoceros unicornis*). The study area has a subtropical climate characterized by hot and humid summers (mid‐March/mid‐June), intense monsoons (mid‐June/mid‐September), and dry autumns/winters (mid‐September/mid‐March) (Lamichhane, Persoon, et al., 2018; Lamichhane, Subedi, et al., 2018). The maximum temperature varies from 35 to 40°C in summer and 14 to 16°C in winter (Jackson, 1994). The mean annual rainfall ranges between 1,138 and 2,680 mm, with over 80% of the rain occurring during the 3 monsoon months (Lamichhane, Persoon, et al., 2018; Lamichhane, Subedi, et al., 2018). Elephants in Nepal are found in four population clusters that is, eastern (Koshi to Jhapa), central (Chitwan to Mahottari), western (Bardiya to Dang), and far‐western (Kanchanpur & Kailali). Elephants frequently migrate along the Nepal–India border through forest connectivity in the Indian region along the east (northern part of West Bengal), west (Uttarakhand), and a few localities in the south (Bihar and Uttar Pradesh).

### 2.2 Elephant attacks data collection

We compiled all available data of elephant attacks on humans (death and injury) from the Divisional Forest Offices (DFO) and Protected Area (PA) offices across the study area for the period 2000 to June 2020. These records are well maintained in the respective DFO and PA offices for providing monetary relief to the elephant‐affected families. Details of the victims and nature of elephant attack (e.g., name, age, sex, address, date of incidence) were obtained from the official records. Each record was verified by reaching at every incident site (locations of the encounter) with the help of victim's spouses, relatives, or neighbors who knew the attack events and locations. We also conducted 30 stakeholder consultation meetings to gather information on human deaths due to elephants, livelihoods, elephant occurrence, and use of areas in and around the village and people's perception toward the elephants.

### 2.3 Victim household questionnaire survey

We conducted structured questionnaire surveys of all affected households (*n* = 412) in the study area. On consent, either the head of the household or another adult member was interviewed. GPS location of each household was recorded. Before an interview, we requested for a verbal consent with the respondents. The questionnaire included the demographic background of the interviewee and the victim (age, sex, ethnicity, family size), socioeconomic status (education, marital status, house type, income source, occupation, land tenure etc.), victim behavior/activity during attack (place of attack, drunk, activities while attacked, methods used for chasing elephant), characteristics of attacking elephant (type of elephant, musth, tusker), and habitat characteristics (land use type) (Table 1).

**TABLE 1:** Variables used in binomial logistic regression and their type/source

Variables	Type of variable	Categories/values	Data source
Elephant characteristics			
Herd type/size	Categorical	Solitary adult bulls, sub‐adult male, sub‐adult male group, herd without calves, female with calves	Questionnaire survey
Musth	Binomial	1, 0, NA (1—Yes, 0—No, NA—Don't know)	Questionnaire survey
Human characteristics			
Response to elephant	Categorical	Shouting, firecracker, stones,	Questionnaire survey
Alcohol use	Binomial	1,0, NA (1—Drunk, 0—not drunk, NA—Don't know)	Questionnaire survey
Victim sex and age	Categorical	Sex (Male, Female) Age (<15, 15—24, 25—44, 45—64, 65+),	Questionnaire survey
Victim ethnicity	Categorical	1. BCT (Brahmin, Chhetri, and Thakuri); 2. Janjati (Ethnic communities of hills and Terai like Gurung, Magar, Tamang, Newar etc.); 3. Indigenous Terai (Tharu, Bote, Darai, Mushahar); 4. *Dalit* (under‐privileged casts of Kami, Damai, Sarki etc.); 5. Madhesi, and 6. Muslim	Questionnaire survey
Education	Categorical	Illiterate, literate, primary, secondary, or above	Questionnaire survey
Activity of the victim at the time of incident	Categorical	Chasing elephants, resting at home, guarding crops, traveling on foot	Questionnaire survey
House type	Categorical	Concrete, CGI sheet, tile house, thatch house	Questionnaire survey
Environmental and habitat characteristics			
Proximity to forest	Numeric		GIS & questionnaire survey
Season	Categorical	Winter, summer, monsoon	Questionnaire survey
Land use type	Categorical	Farmland, settlements, forests/grassland	GIS

Note

The human casualty in elephant attack was the dependent variable, and the independent variables included elephant characteristics, human characteristics, and environmental and habitat characteristics.

### 2.4 Data analysis

We entered all the questionnaire survey data in MS Excel and prepared descriptive summaries using pivot table function (Dan Clark, 2020). We then performed data analyses in the R statistical package v. 4.0.2 (R Development Core Team, 2020). We used chi‐square test of independence with the null hypothesis—there was no any significant difference on frequency of attacks (death and injury) among different districts, seasons, months, ethnicity, age group, sex, and occupation of the victim (Lamichhane, Persoon, et al., 2018). We categorized victims into five categories based on ethnicity, upper caste Hindus including Brahmin Chhetri Thakuri (BCT), *dalit* or socially disadvantaged marginal communities, *Janajati* (ethnic groups such as *Gurung*, *Magar*, *Newar*, *Tamang*, *Rai*, *Limbu*, *Tharu*, *Bote*, *Darai*, *Rajbansi* etc.), Madhesi, and Muslim. Similarly, we grouped the victims into five age categories, that is, <15, 15–24, 25–44, 45–64, and 65+ years following (United Nations, 1982). Education level of the victims was categorized into illiterate (who cannot read and write), literate (who can read/write but have not attended formal school), primary (completed primary school), and secondary or above. Housing of the victim was categorized into cemented house, corrugated galvanized iron (CGI) sheet roof house, tiled roof house, and thatched house.

We carried out binomial logistic regression by constructing a generalized linear mixed model (GLMM) (Zuur et al., 2010) to determine the factors associated with fatalities in elephant attacks. In the GLMM, fatalities on elephant attack were used as dependent variable by coding the human fatality—1 and injury—0. Fourteen explanatory variables representing elephant characteristics, human characteristics, and site characteristics were defined (Table 1). Elephant behavior included social characteristics (solitary bulls or herd elephant), and the elephant was in *musth*. The human characteristics included age and sex of the victim, education, activities of the victim during elephant attack, location of attack, and type of house of victims. Human behavior or response toward elephants (chasing with fire, explosives, or gun) was also included. Site characteristics included place of attack, migration route of elephants, and proximity to forest. We extracted the victim location's habitat and environmental variables (Naha et al., 2019) (Table 1) using Google earth engine platform (Buchholtz et al., 2020; Gorelick et al., 2017) and Arc‐GIS v 10.5 (ESRI, 2016; Wang et al., 2018).

We ranked models by the small‐sampled corrected Akaike's information criteria (AICc, lower AICc value indicates higher model ranking) using multi‐model inference in “MuMIn” package in R (Barton, 2020). The top model for making ecological inference was obtained by averaging the models in the candidate set supporting the data equally well (AICc ≤ 2, (Burnham & Anderson, 2001).

## 3 RESULTS

### 3.1 Victim characteristics

There were 412 records (274 fatalities and 138 injuries) of elephant attacks on humans for the period of 2000–2020 June. Men were attacked more frequently than women. Ethnic or *Janajati* people were the most affected group followed by BCT, *socially disadvantaged, Madhesi*, and Muslim. Age of the victims on elephant attacks range from 7 months to 80 years but most of them (71%) were adults between 25 and 64 years (Table 1). A quarter of elephant attacks occurred while people were chasing elephants and half took place around settlements or homes (Table 2). Most of the people attacked (88.8%) had low level of education (illiterate, literate, or primary education only), and the two third of the victims of elephant attacks were living in the thatched house (Table 3).

**TABLE 2:** Characteristics of victims attacked by elephants in Nepal's Terai and Chure region of Nepal between 2000 and June 2020

Victim characteristics	Incident type		Total
	Death	Injury	
Sex			
Women	116	38	154
Men	158	100	258
Caste/ethnicity			
BCT	74	49	123
Dalit	46	20	66
Janajati	115	50	165
Madhesi	36	15	51
Muslim	3	4	7
Age			
<15	19	7	26
15–24	39	23	62
25–44	101	61	162
45–64	92	39	131
65+	23	8	31
Education			
Illiterate	141	63	204
Literate	44	36	80
Primary	55	27	82
Secondary or above	34	12	46
Housing			
Cemented house	28	22	50
CGI sheet roof house	31	27	58
Tiled roof house	28	9	37
Thatched house	187	80	267
Total	274	138	412

**TABLE 3:** Victim activity and location of elephant attacks in the Terai and Chure region of Nepal during 2000–June 2020

Activity of the victim	Location of attack			Total
	Crop field	Forest	Home/settlement	
Chasing elephants	11	22	70	103
Traveling	1	30	50	81
Sleeping or working at home	–	–	66	66
Fetching forest products	–	65	–	65
Guarding crops	36	1	2	39
Livestock grazing	2	23	1	26
Open defecation	–	–	21	21
Other	1	7	3	11
Total	51	148	213	412

### 3.2 Elephant characteristics

Most of the elephant attacks on humans (85.2%, *n* = 412) were caused by solitary adult bulls or subadult male groups and rest of the attacks were caused by the elephants in herd or females separated from the herd (Table 4). The bulls involved in the attacks were in *musth* in more than half of the incidents.

**TABLE 4:** Characteristics of the elephants involved in attacks on humans in Nepal's Terai and Chure region between 2000 and June 2020

Elephant characteristics	Attacks on humans		Total
	Death	Injury	
Group type			
Adult males	213	103	316
Adult females	6	13	19
Mixed group herd	17	11	28
Subadult male group	27	8	35
Unknown	11	3	14
Adult/subadult bull elephant			
Yes	240	111	351
No	24	24	48
Don't know	10	3	13
Elephant in *musth*			
Yes	131	76	207
No	71	36	107
Don't know	72	26	98
Total	274	138	412

### 3.3 Temporal and spatial distribution of elephant attacks on humans

**FIGURE 3:**
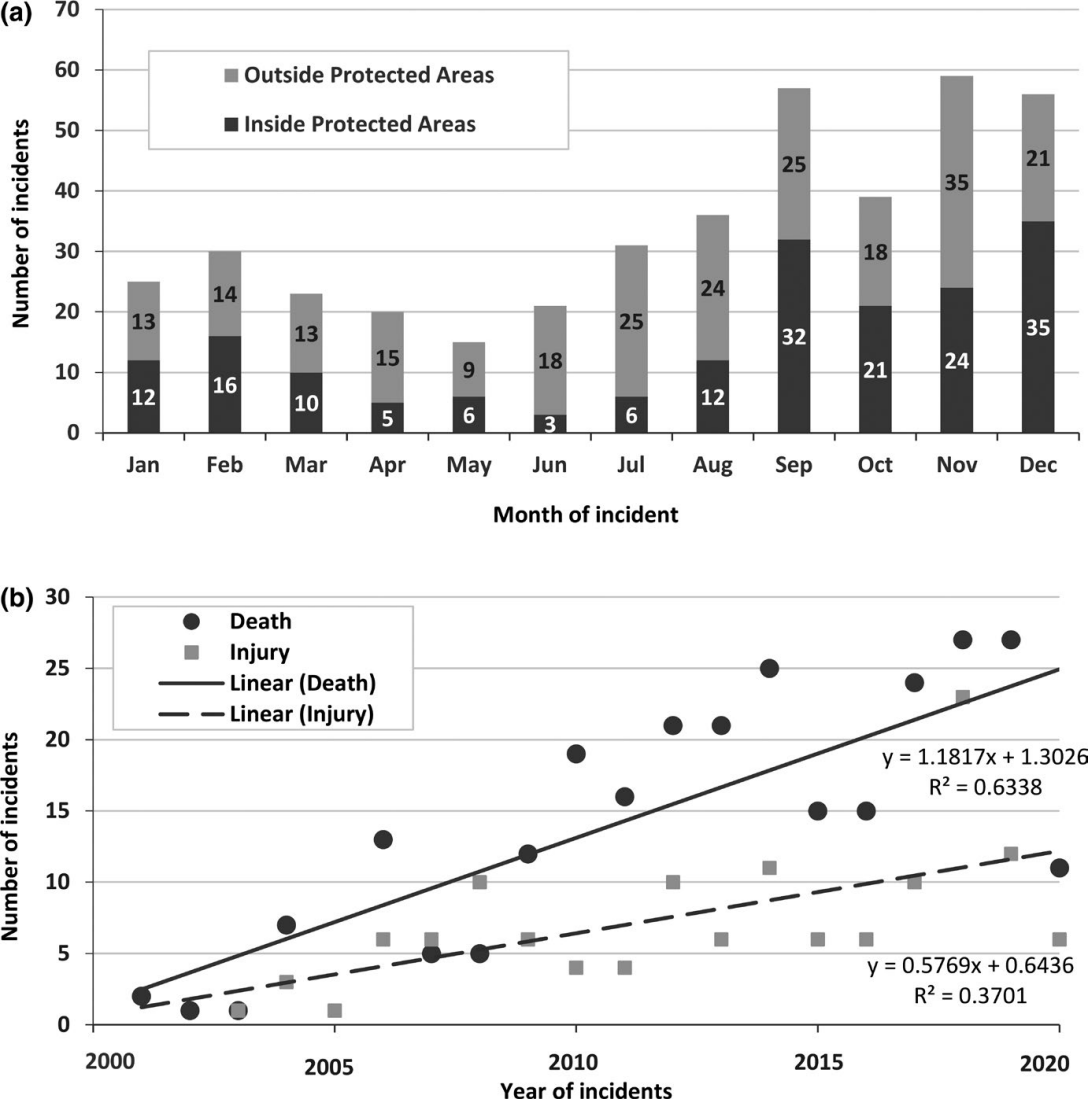
Temporal distribution of elephant attacks on humans (death and injuries) in Nepal during 2000 and June 2020 (a) over the months and (b) years

Elephant‐related human attacks varied significantly across months (*χ* ^2^ = 76.272, *df* = 11, *p* < .001) with peak during postmonsoon season (September to December) (Figure 3a). Number of elephant‐related human attacks were higher outside protected areas (Figure 5), but the difference was not significant (*t* = −1.0751, *df* = 19.296, *p* = .29). Linear regression showed a gradual increase in elephant attacks on humans (Figure 3b). Average number of incidences of attacks was 11 (±8.5*SD*) during 2000–2010 and increased to 29 attacks (±11.2*SD*) during 2011–June 2020.

**FIGURE 4:**
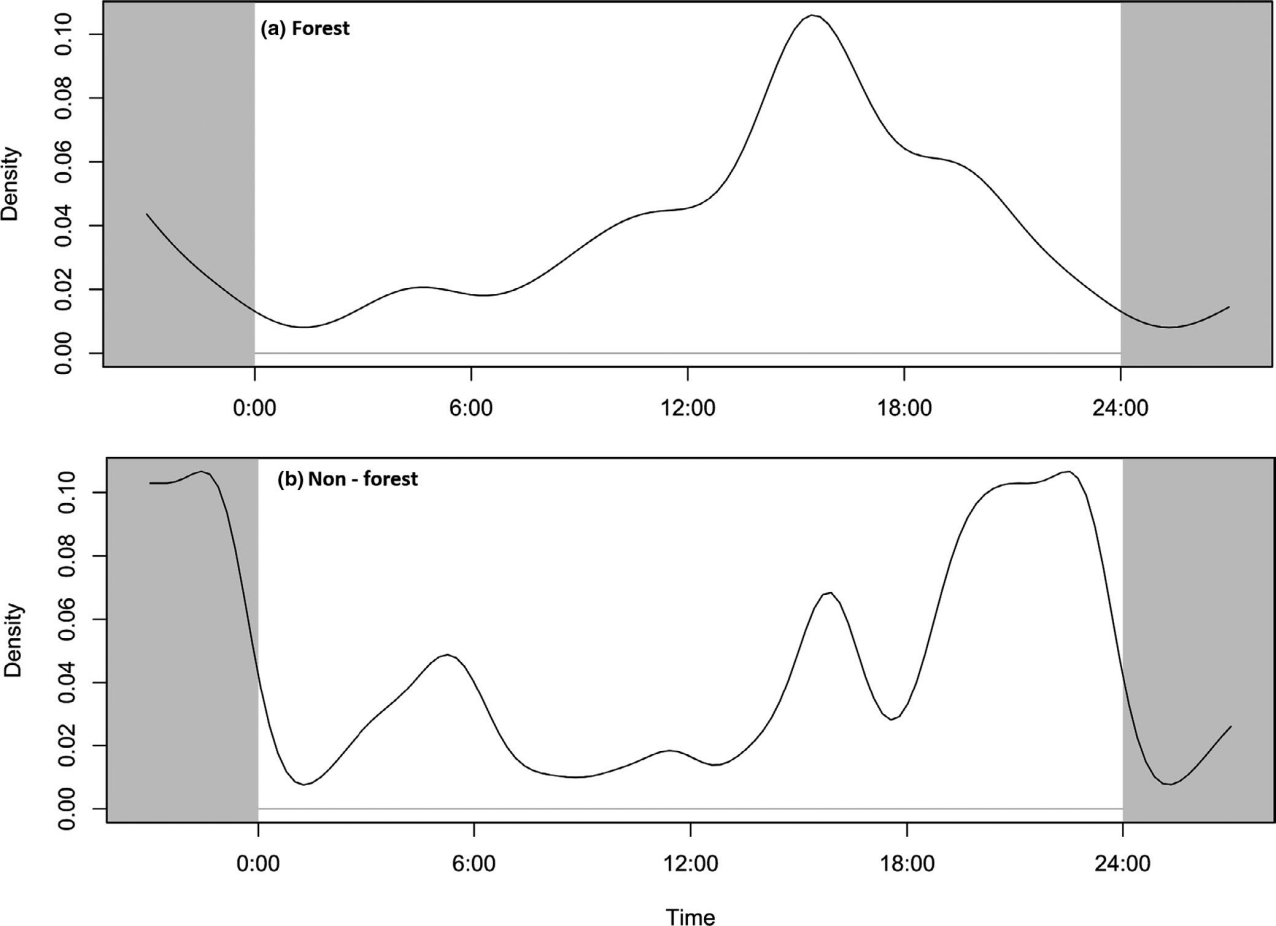
Elephant attacks on humans at the different time of day in (a) forested areas and (b) settlement and agriculture areas outside forests

In the forested areas, elephant attacks on humans were at peak in the afternoon (4–5 p.m.), whereas, in settlement areas, elephant attacks peaked in the evening (7–9 p.m.) (Figure 4).

**FIGURE 5:**
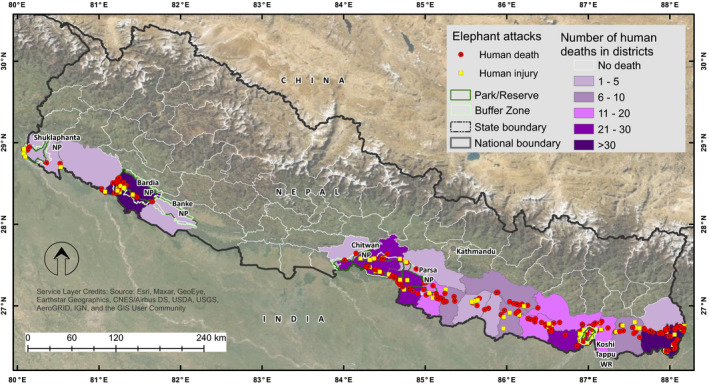
Spatial distribution of elephant attacks on humans in Nepal

**FIGURE 6:**
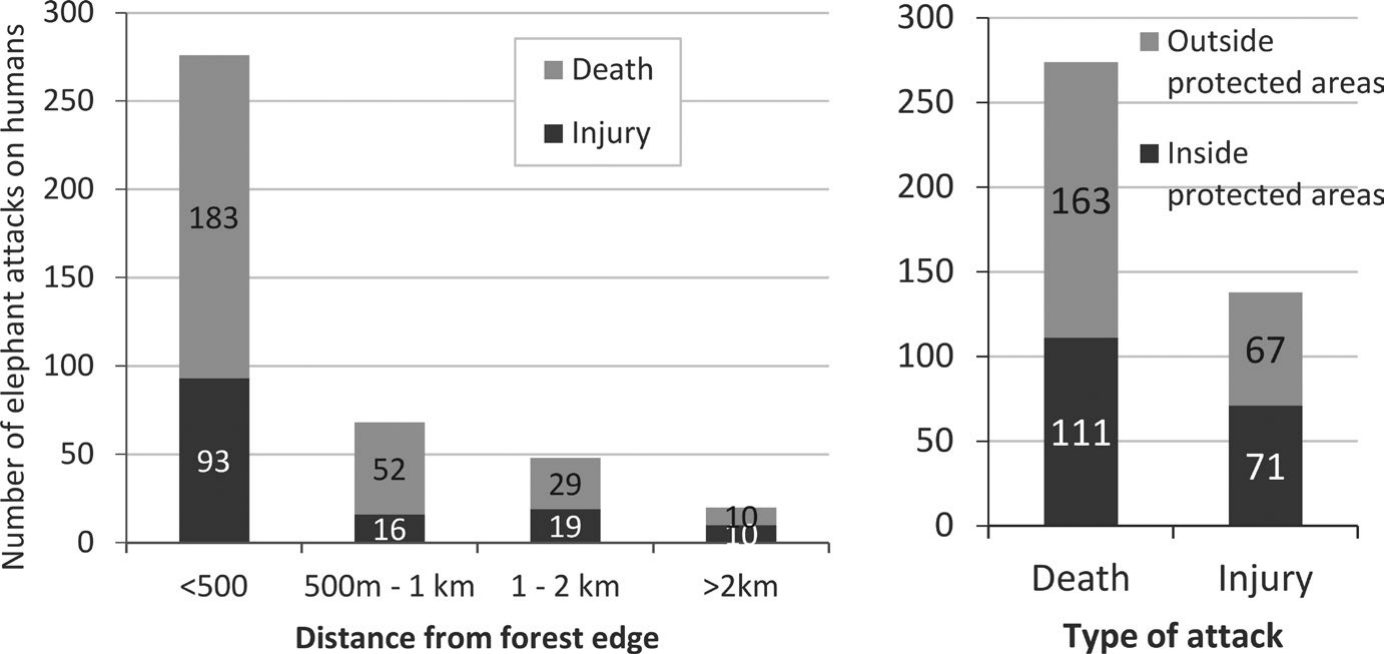
Spatial distribution of elephant attacks on humans in Nepal with respect to distance from forest edge (left) and inside/outside of the protected areas (right)

The number of attacks on humans varied significantly among the districts (*χ* ^2^ = 338.49, *df* = 19, *p*‐ < .01) with the highest number of incidents (*n* = 66) from Jhapa and Bardiya districts (Figure 5). The majority of elephant attacks (67%) occurred within 500 m from the forest edge (Figure 6).

### 3.4 Factors associated with human fatality

People who were drunk and chasing elephants using firecrackers were more vulnerable to fatalities while chasing elephants using fire was negatively associated with fatalities (Table 5).

**TABLE 5:** Factors associated with human fatalities on elephant attacks in Nepal

Parameters	Estimate	*SE*	Adjusted *SE*	*z* value	Pr(>|*z*|)	Significance
(Intercept)	0.652	0.795	0.798	0.818	0.413	
Crackers_Drums	1.095	0.508	0.511	2.142	0.032	^*^
Drunk	1.124	0.380	0.382	2.938	0.003	^**^
Fire_chasing	−1.715	0.576	0.579	2.961	0.003	^**^
House_typeCGI	0.063	0.588	0.592	0.107	0.915	
House_typethatched	0.795	0.504	0.508	1.566	0.117	
House_typetiled	1.585	0.828	0.833	1.903	0.057	.
Place_attackForest	−0.914	0.530	0.533	1.715	0.086	.
Place_attackHome/settlement	−0.272	0.522	0.526	0.518	0.605	
prox_forest	−0.001	0.000	0.000	1.919	0.055	.
Age	0.013	0.010	0.010	1.219	0.223	
Ele_Musth	−0.294	0.358	0.360	0.816	0.414	

Note

Significance codes: <0.001 “***”0.001 “**” 0.01 “*”0.05“.”0.1 “ ” 1.

## 4 DISCUSSION

Our study presents the most comprehensive analysis of the elephant attacks on humans in Nepal. Elephants attacked an average of 20 humans per year with two thirds resulting into fatalities in the Terai and Chure region. We documented the increasing trend of Elephant attacks on humans over the years. Human response toward elephants was a major factor resulting in elephant attacks, supporting our first hypothesis. Higher number of attacks by elephants on humans occurred in proximity of the forest (66.9% attacks in <500 m from forest edge) supporting our second hypothesis. In contrast to our third hypothesis, more people were attacked outside the protected areas. Over 76% of the attacks on humans were caused by the solitary bulls supporting our third hypothesis.

### 4.1 Characteristics of the victims of elephant attack

Elephants attacked men more frequently than women which can be associated with the high mobility of males and their involvement in chasing the elephants (Sarker et al., 2015). For instance, majority of the males were attacked while chasing elephants or traveling whereas females were attacked more frequently while fetching forest products or working at home (Supplementary information S1). Most of the attacks on humans occurred close to forests where socio‐economically marginalized people reside (Neupane et al., 2017; Pant et al., 2016). Marginalized communities refer to the people or communities at the lower level of the society with limited access to economic opportunities and the social decision‐making. Mostly, they live in vulnerable areas such forest encroachment, river sides, and roadsides. Such communities have often excluded from the mainstream activities (economic, political, cultural, and social) of the society and less access to the government resources. Most of the attacked persons were illiterate, living in thatched house, an indicator of poor social and economic condition (Neupane et al., 2013). People living in thatched house often keep their grain storage close to where they sleep due to limited space in the house. It increases the chances of elephant damage in their house and risks of elephant attack (Naha et al., 2019). Neupane et al. (2013) documented low level of education and awareness about elephants as an important determinant of the elephant attacks on humans. High proportion of attacks on *Janajati* (ethnic) people can be associated with their involvement in local liquor production, consumption, and selling for their livelihood (Lamichhane, Subedi, et al., 2018; Parajuli et al., 2015). Such liquor also attracts elephants (Naha et al., 2019), primarily the solitary bulls, and increases the chances of encounter with humans. Thus, people living in the settlements near to the forest edge, especially on the elephant migration routes, are vulnerable to elephant attacks (Jadhav & Barua, 2012; ten Velde, 1997). The findings of this study clearly indicate a need of devising strategies and actions to stop elephants entering into the settlements and help poor and socially marginalized communities to secure their livelihoods (Gross et al., 2021).

### 4.2 Characteristics of elephants attacking humans

Mixed herd elephants rarely attacked humans (<5% of the incidents) although they are involved in crop raiding during migration through agriculture areas or settlements (Naha et al., 2020). Solitary adult bull elephants caused majority of attacks on humans in Nepal (Acharya et al., 2016) but it varied among elephant individuals. A few individual bulls that repeatedly visited human settlements and agriculture areas were involved in the majority of the attacks. Similar findings of attacks on humans by solitary bulls are reported from some parts of Bangladesh (Sarker et al., 2015). In general, bulls range widely, operate solitarily or in small groups and young bulls that disperse out of natal herds lack the social buffering that herd individuals would have. Thus, young bulls can be more excitable and due to their wide movement, bulls can come into frequent contact with people, some of which can turn severely negative (Fernando et al., 2008). However, they are also harassed by people most of the time while raiding crops or grain stores. These irritating actions of humans make them more aggressive resulting in violent attacks (Sampson et al., 2019). We identified 37 such bulls causing three quarters of all attacks on humans in the last twenty years, some of them caused a disproportionately higher number of attacks (up to 36). Such individuals can be termed “problem individuals” need to be closely monitored, particularly their movement patterns and ranging behavior (Lamichhane et al., 2017). The knowledge thus gained through monitoring can be helpful in prioritizing appropriate management strategies.

### 4.3 Temporal patterns of elephant attacks

Documented records of elephant attacks on humans in Nepal goes back to the 1970s (B.N. Uprety, 2020, Personal Communication.) with sporadic records until the late 1990s (Smith & Mishra, 1992). In our study, we only included data between 2000 and June 2020. The wild elephant population has gradually increased in Nepal from 52–53 individuals during the 1990s (Smith & Mishra, 1992) to 107–145 individuals in 2007 (DNPWC, 2009) and >200 individuals in 2020 (Ram & Acharya, 2020). Human population growth rate in the Terai and Chure region (1.72%) is also higher compared with the national average (1.35%; (CBS, 2012)). Consequently, the deforestation rate is also higher in this region especially in the Chure (0.18% annually) (DFRS, 2015). The remaining forests are also becoming increasingly fragmented with planned and ongoing large‐scale infrastructure development such as roads, railways, canals, industries, airports, and urban areas forming barriers to elephant migration (MOFSC, N, 2015). Overlap in forest use by elephants and humans is increasing, resulting a high human–elephant interactions (Acharya et al., 2016; Lamichhane, Persoon, et al., 2018; Mariki et al., 2015; Mukeka et al., 2019).

Elephant attacks on humans occurred throughout the year but peaked during September–December coinciding with the rice harvesting season. Lamichhane, Persoon, et al. (2018) also shows that elephants use both forested and human‐dominated areas but use of human‐dominated areas varies seasonally with peak in the autumn. Premonsoon (March–June) had the lowest level of attacks as agriculture areas are devoid of crops and elephants are concentrated primarily in the forests feeding on natural vegetation (Koirala et al., 2016; Lamichhane, Persoon, et al., 2018).

Elephant attacks inside forests peaked during the afternoon (~16:00) when human activity, mainly cattle grazing, and fodder and forest resource collection would remain high inside the forests. The elephants generally rest during the mid‐day hot period and start become active with decreasing temperature in the afternoon (after 15:00) (Thapa et al., 2019). This increases the chance of interaction between elephants and humans. Close to the time of sunset (~18:00), most of the people are returning home from the forest while elephants remain inside forest so decreasing the chances of interaction between them. Elephant attacks again increase in the evening (19:00–21:00) when elephants enter the settlements or agricultural fields and people come in direct confrontation while chasing elephants away. Changing behavior of people through conservation education, restoration of the elephant movement corridors and bottlenecks, devising elephant and people friendly land use policies will contribute to human–elephant coexistence.

### 4.4 Spatial pattern of elephant attacks

Two‐thirds of elephant attacks on humans occurred within 500 m from the forest edge. People living in proximity of forests are vulnerable to elephant attacks because (a) chances of encountering elephants are high at close distance to forest, (b) generally economically marginalized communities live in these areas with lack of proper housing (thatched houses), which is often flimsy. Similar finding of a higher number of attacks by wildlife close to forest or park boundary (<1 km) and an inverse relationship between the distance from the forest edge and wildlife attacks is reported in other studies (Gurung et al., 2008; Lamichhane, Subedi, et al., 2018; Pant et al., 2016).

A higher number of elephant attacks outside protected areas (59.5%) in our study is consistent with Acharya et al. (2016). Similar results with higher conflict incidents outside protected areas have been reported from north‐east India as well (Choudhury, 2004). Elephants require large areas to meet their nutritional and social needs. In fragmented habitats where the forests have become insular in human‐use areas, elephant home ranges are not limited to protected areas, but encompass human‐use areas as well. People living close to protected areas are aware of elephant behavior and respond accordingly (Lamichhane et al., 2019). However, beyond protected areas, human response toward elephants is more aggressive due to low level of awareness, resulting in a high number of human casualties as well as retaliatory killing of elephants (25 out of 33 retaliatory killing in past 20 years, unpublished data compiled by the first author).

The elephant attacks on humans were concentrated in four pockets, Jhapa, Koshi, Chitwan Parsa, and Bardiya. Despite the smaller population of elephants (~35) in eastern Nepal, the number of attacks on humans is relatively higher (43% of total attacks in Nepal). The reason for such a high casualty in eastern Nepal especially in Jhapa district of south‐eastern border of Nepal is because of (a) the highly fragmented habitats outside of the protected areas, (b) severe disruption in the historical migratory corridors of elephants from West Bengal, India, straddling the national boundary, and (c) incompatible human behavioral response toward elephants in interface areas where interactions have become common. Historically, ~100 elephants used to migrate annually from West Bengal (India) entering Nepal from the eastern border during September–October and May–June (Mallick, 2012). While migrating, they often came in confrontation with people as they are forced to travel through settlements and agricultural land, with a large part of their historic migration route encroached by people (Choudhury, 2004). A fence installed in Bahundangi area (Jhapa district) at the Eastern border of Nepal has contributed to reducing human–elephant conflict in the fenced areas (Naha et al., 2019). However, the elephant continues their movement in Nepal from south of the fenced area (NTNC, 2019). Some elephants, especially males, break the fence and continue their movement up to Koshi Tappu Wildlife Reserve (KTWR) and westwards.

A quarter of all elephant attacks on humans in Nepal occurred in KTWR and its periphery. KTWR acts as a stepping stone for the elephant population in eastern Nepal. The KTWR (173 km^2^) is much smaller than the home range of elephants (188–400 km^2^, Williams et al., 2008; Alfred et al., 2012; Williams et al., 2015). With the high dependency of communities on the reserve for grazing, fodder, firewood, and fishing, elephants and people come in frequent confrontation. The situation is further aggravated in the densely populated agrarian areas in the periphery of the reserve.

Human casualty was recorded throughout Central (up to Nawalparasi East) and Eastern Terai. In the Chitwan‐Parsa Complex in central Nepal, 27.4% of the elephant attacks were recorded, mostly from Chitwan, Parsa, and Bara districts. There is a gap in elephant distribution between the central population (Nawalparasi East) and the western population (Bardiya) with only a sporadic presence in Banke, Dang, and Kapilvastu districts (Lamichhane, Persoon, et al., 2018). The largest elephant population (>100) in Nepal exists primarily in Bardiya NP in western Nepal where 16.7% of total elephant attacks on humans occurred. Elephants in the western population also migrate through the Chure foothills west of Bardiya reaching up to Shuklaphanta NP causing some incidents of attacks on humans (ten Velde, 1997).

### 4.5 Factors associated with the human fatality

Our results of two third of elephant attacks resulting in the fatality are consistent with Acharya et al. (2016). Human behavior and responses toward elephants were the major factors to cause elephant attacks on humans. Aggressive human behavior toward elephant with intolerance was the major determinant of human fatality in elephant attack (Nelson et al., 2003). People were killed mostly while chasing wild elephants using firecrackers and other high sound and light objects. Such elephant drives when haphazardly done would increase the fear and trigger defensive offensive behavior in elephants increasing the probability of human attacks. In the Terai, consumption of alcoholic beverages by local communities during the leisure hours of evening and night is high. Driving elephants when they come to crop fields in an inebriated state can increase the vulnerability of attacks by elephants (Neupane, et al., 2017). Negative association of fatalities while chasing elephants using fire torch indicates it as a safe and effective method for pushing elephants outside of the village.

## 5 CONCLUSIONS

Human casualties from elephants have been increasing with its multifaced impact on human–elephant coexistence in Nepal. Elephant attacks were concentrated in proximity of forests primarily affecting the socioeconomically marginalized communities. Most of the attacks on humans were caused by solitary bull elephants. Human response toward elephant was a major factor associated with the elephant attacks on humans. Chances of elephant attacks and human fatalities increase when drunk people are chasing elephants. Local people as well as the Government of Nepal (GON) have adopted various preventive and curative measures such as fences in hotspots, problem animal management, and relief support for victims/families to reduce both human casualties and elephant retaliation. These measures should be continued and additional activities such as integrated settlement, safe housing for socioeconomically marginalized community, and community grain house in the settlement could be promoted to reduce the confrontation between elephants and humans. Revising the “elephant conservation action plan and strategy for Nepal” and its effective implementation will promote human–elephant coexistence HECx and sustain increasing elephant populations of Nepal.

## CONFLICT OF INTEREST

None declared.

## AUTHOR CONTRIBUTIONS

**Ashok Kumar Ram:** Conceptualization (equal); data curation (equal); formal analysis (equal); funding acquisition (equal); methodology (equal); project administration (equal); writing–original draft (equal); writing–review and editing (equal). **Samrat Mondol:** Conceptualization (equal); formal analysis (equal); supervision (equal); writing–review and editing (equal). **Naresh Subedi:** Conceptualization (equal); funding acquisition (equal); supervision (equal); writing–review and editing (equal). **Babu Ram Lamichhane:** Data curation (equal); formal analysis (equal); methodology (equal); supervision (equal); validation (equal); writing–original draft (equal); writing–review and editing (equal). **Hem Sagar Baral:** Supervision (equal); writing–review and editing (equal). **Laxminarayanan Natarajan:** Methodology (equal); writing–review and editing (equal). **Rajan Amin:** Methodology (equal); writing–review and editing (equal). **Bivash Pandav:** Conceptualization (equal); supervision (equal); writing–review and editing (equal).

## ETHICAL APPROVAL

We obtained research permissions from the Department of National Parks and Wildlife Conservation Nepal (Ref no: 3066/073/74; June 02, 2017). We did not carry out any experiments with live animals. We only carry out stakeholder consultation and questionnaire survey by taking verbal consent from the participants. We removed individual information of participants prior to analyze data to protect the participants’ privacy.

## Supporting information

Supplementary Information

## Data Availability

Data related to this article are deposited in Dryad and are available via the following link. https://doi.org/10.5061/dryad.fxpnvx0s1.
