# Supplementary material for: Patterns and determinants of elephant attacks on humans in Nepal
Source: Ecol Evol. 2021 Aug 1;11(17):11639–50. doi: 10.1002/ece3.7796 (PMC8427586; doi:10.1002/ece3.7796)
Supplement: Supplementary file 1 — Supplementary Information [file ECE3-11-11639-s001.docx]

Supplementary Information S1. Sex of victim and their activity while attacked by elephant.

| Activity of the victim | Death | | Injury | | Total |
| --- | --- | --- | --- | --- | --- |
|  | female | male | female | male |  |
| Chasing elephants | 14 | 51 | 13 | 25 | 103 |
| Fetching forest products | 23 | 20 | 14 | 8 | 65 |
| Guarding crops | 7 | 23 | 3 | 6 | 39 |
| Livestock grazing | 5 | 12 | 4 | 5 | 26 |
| Open defecation | 6 | 5 | 4 | 6 | 21 |
| Other | 1 | 7 | 1 | 2 | 11 |
| Sleeping/working at home | 27 | 22 | 5 | 12 | 66 |
| Travelling | 16 | 35 | 10 | 20 | 81 |
| Grand Total | 99 | 175 | 54 | 84 | 412 |
